# Effect of *Chlorella Pyrenoidosa* Protein Hydrolysate-Calcium Chelate on Calcium Absorption Metabolism and Gut Microbiota Composition in Low-Calcium Diet-Fed Rats

**DOI:** 10.3390/md17060348

**Published:** 2019-06-11

**Authors:** Pengpeng Hua, Yu Xiong, Zhiying Yu, Bin Liu, Lina Zhao

**Affiliations:** 1 National Engineering Research Center of JUNCAO Technology, Fujian Agriculture and Forestry University, Fuzhou 350002, China; huapengpeng_flower@163.com (P.H.); aindxiong@163.com (Y.X.); 18838018055@163.com (Z.Y.); 2 College of Food Sciences, Fujian Agriculture and Forestry University, Fuzhou 350002, China

**Keywords:** *Chlorella pyrenoidosa* protein hydrolysate (CPPH), *Chlorella pyrenoidosa* protein hydrolysate-calcium chelate (CPPH-Ca), calcium absorption, gene expression, gut microbiota

## Abstract

In our current investigation, we evaluated the effect of *Chlorella pyrenoidosa* protein hydrolysate (CPPH) and *Chlorella pyrenoidosa* protein hydrolysate-calcium chelate (CPPH-Ca) on calcium absorption and gut microbiota composition, as well as their in vivo regulatory mechanism in SD rats fed low-calcium diets. Potent major compounds in CPPH were characterized by HPLC-MS/MS, and the calcium-binding mechanism was investigated through ultraviolet and infrared spectroscopy. Using high-throughput next-generation 16S rRNA gene sequencing, we analyzed the composition of gut microbiota in rats. Our study showed that HCPPH-Ca increased the levels of body weight gain, serum Ca, bone activity, bone mineral density (BMD) and bone mineral content (BMC), while decreased serum alkaline phosphatase (ALP) and inhibited the morphological changes of bone. HCPPH-Ca up-regulated the gene expressions of transient receptor potential cation V5 (TRPV5), TRPV6, calcium-binding protein-D9k (CaBP-D9k) and a calcium pump (plasma membrane Ca-ATPase, PMCA1b). It also improved the abundances of *Firmicutes* and *Lactobacillus*. *Bifidobacterium* and *Sutterella* were both positively correlated with calcium absorption. Collectively, these findings illustrate the potential of HCPPH-Ca as an effective calcium supplement.

## 1. Introduction

As one of the most abundant mineral elements in human body, calcium plays a critical role in human bone health, especially for children [1] and the elderly [2]. A low intake and bioavailability of calcium may cause calcium deficiency [3], which is characterized by low levels of calcium and alkaline phosphatase (ALP) in serum, as well as low bone mass. Calcium deficiency causes microarchitectural deterioration of bone tissue, leading to increased bone fragility and risk of fracture [4,5,6]. Considerable efforts have been devoted to developing appropriate treatments because of the medical importance of calcium deficiency. Although many calcium supplements are available on the market, their efficacies are often low and side effects are common [7]. Calcium gluconate (HGCa), inorganic calcium (CaCO_3_) and calcium lactate are the main forms of ionized calcium in the intestinal environment, which have the disadvantage of easily forming calcium phosphate deposition [7]. As a result, the bioavailability and the absorption of dietary calcium is severely lowered [8]. Therefore, a well-tolerated treatment with minimal side effects for calcium absorption is urgently required. Many calcium-containing complexes and commercial products are currently available to prevent calcium deficiency in humans [9]. Previous studies have shown that substances such as casein phosphopeptides (CPPs) and phosvitin phosphopeptides (PPPs) can improve calcium absorption [10,11,12]. In addition, soybean, fish bone, hen egg white peptides (EPs), shrimp processing by-products and whey protein have also been shown to facilitate the in vivo calcium absorption, while their in vivo effect remains largely unclear [13,14,15,16,17,18,19,20].

*Chlorella pyrenoidosa* is a genus of unicellular green algae which contains many substances that could be beneficial for human health, such as proteins, β-carotene and amino acids [21,22,23]. It is a good material for biotechnology research, and is also a source of high-quality single-cell protein [24,25,26,27]. At present, active peptides derived from *Chlorella pyrenoidosa* proteins are research hotspots worldwide. Most studies focus on the development of functional peptides, such as *Chlorella pyrenoidosa* protein (CCP), which has antioxidant, antimicrobial, blood pressure-reducing, lipid-lowering and immune regulatory properties [28,29,30,31,32,33]. However, there is almost no research on the activity of *Chlorella pyrenoidosa* calcium-chelating peptide.

At the molecular level, transient receptor potential cation V5 (TRPV5) [34,35,36], TRPV6 [37,38], calcium-binding protein-D9k (CaBP-D9k) and a calcium pump (plasma membrane Ca-ATPase, PMCA1b) [39,40,41,42,43,44,45,46] can modulate calcium re-absorption in the renal tubules [47,48]. It is widely accepted that gut microbiota has beneficial effects on calcium absorption and bone health. Some bacterial genera, such as *Bifidobacterium* and *L. reuteri,* are positively correlated with levels of serum Ca, ALP, bone mineral content (BMC) and bone mineral density (BMD) [49]. Moreover, Marine algae can regulate the composition of microbiota [50] and have a beneficial effect on the improvement the Ca absorption [51]. However, the calcium-promoting mechanism of *Chlorella pyrenoidosa* protein hydrolysate-calcium chelate (CPPH-Ca) has not been well studied. Therefore, in the present study, we aimed to assess the potential calcium absorption of HCPPH-Ca in rats fed low-calcium diets. Furthermore, we also determined the specific gene expression and the composition of gut microbiota.

## 2. Results

### 2.1. Characterization of Potent Major Compounds of CPPH and Structural Characterization of CPPH-Ca

Table S1 illustrates the identified peptide sequences of CPPH. A total of 43 peptide sequences were detected from CPPH. Retention times ranged from 4.21 min to 8.37 min. Figure S1 shows that the ultraviolet absorption spectrum of CPPH and CPPH-Ca demonstrated obvious band shifts. When the CPPH and Ca ions chelated, the UV absorption spectra of CPPH obviously shifted/changed both in band and intensity in the area of 223 to 274 nm. The absorption peak of WPH shifted from 230 to 265 nm. The CPPH-Ca presented distinct UV absorption spectra compared with CPPH, suggesting that the new substance was formed when CPPH interacted with calcium ions. Figure S2 shows that the FT-IR spectrum curve of CPPH-Ca was different from CPPH. CPPH-Ca had obvious fluctuations at 3410 cm^−1^, 1650 cm^−1^ and 1400 cm^−1^, while CPPH exhibited obvious absorption peak at around 3350 cm^−1^, 1642 cm^−1^ and 1476 cm^−1^. Meanwhile, the spectra of the CPPH showed two strong bands at 1642 cm^−1^ and 1625 cm^−1^(amide-I). After binding with calcium, two peaks appeared at 1650 cm^−1^ and 1400 cm^−1^, with that at 1400 cm^−1^ showing the symmetric stretching vibration of -COO. The results indicate that the interaction site between calcium and CPPH is carboxyl oxygen.

### 2.2. Body Weight and Biochemical Parameters in Serum

Table 1 lists the changes in the body weights of rats in different groups. During the experimental period, no rats died. At week 0, no significant difference in terms of initial body weight was observed among the 10 groups. After 4 weeks, the body weight of low calcium diet-fed (model) was obviously lower compared with the normal diet-fed (control) group. In addition, after 8 weeks, the rats fed with HCPPH-Ca in the high dose group gained body weight more rapidly compared with the model group and HCaCO_3_ (*p* < 0.05). However, there was no significant difference between the HCPPH-Ca group and control group. After 8 weeks, the final body weight gain of the MCPPH + MCaCO_3_, HCPPH + HCaCO_3_, MCPPH-Ca and HCPPH-Ca groups was significantly higher than that of the model group (*p* < 0.05). These results indicated that CPPH-Ca could effective improve body weight to a normal level, even in the middle dose group, that HCPPH-Ca was better than HCaCO_3_, even in HGCa, and that calcium in CPPH-Ca was more easily to be absorbed.

**Figure 1:**
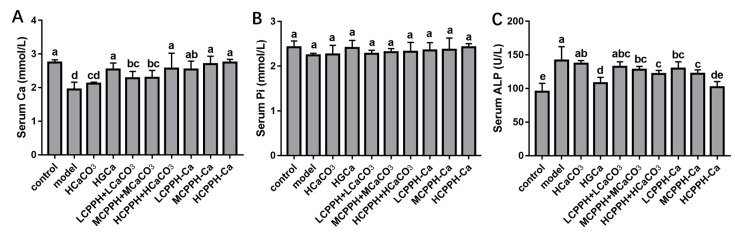
Biochemical parameters in the serum of Ca-deficient rats after oral gavage with different Ca. Serum Ca (**A**), Serum P (**B**), Serum alkaline phosphatase (Serum ALP) (**C**). Note: control, normal group; model, low calcium group; HCaCO_3_, high dosage of CaCO_3_ group; HGCa, high dosage of calcium gluconate group; LCPPH + LCaCO_3_, low dosage of CPPH supplemented with low dosage of CaCO_3_ group; MCPPH + MCaCO_3_, model dosage of CPPH supplemented with model dosage of CaCO_3_ group; HCPPH + HCaCO_3_, high dosage of CPPH supplemented with high dosage of CaCO_3_ group; LCPPH-Ca, low dosage of CPPH-Ca group; MCPPH-Ca, model dosage of CPPH-Ca group; HCPPH-Ca, high dosage of CPPH-Ca group. Data are expressed as the mean ± SD (*n* = 10). One-way ANOVA with Tukey’s test. Different letters indicate significant effect with *p* < 0.05.

Figure 1 summarizes the changes about the biochemical parameters in the serum. After 8 weeks, the serum phosphorus (p) level was not significantly changed under different treatments (*p* < 0.05) (Figure 1B). However, rats fed with the serum Ca level increased significantly in control group compared with the model group (*p* < 0.05) (Figure 1A), the serum ALP activity were decreased significantly in control group compared with the model group (*p* < 0.05) (Figure 1C). Particularly, rats fed with supplements at low, medium or high dose of CPPH-Ca and high dose of CPPH + CaCO_3_ showed a significant increase of serum Ca level compared with the model group even CaCO_3_ group (*p* < 0.05), while those groups were no different significantly from control group and HGCa group. The ALP activity in the serum showed no significant difference between the HCPPH-Ca and control groups (Figure 1C), while those groups showed a significant decrease in ALP compared with the model group (*p* < 0.05). A high dosage of CPPH + CaCO_3_ and CPPH-Ca deduced a greater ALP activity compared with low dosages of CPPH + CaCO_3_ and CPPH-Ca. Therefore, these results reveal that CPPH-Ca, even in the low dose group, was more easily absorbed and calcified.

### 2.3. Bone Biomechanical Parameters and Histomorphometry

**Figure 2:**
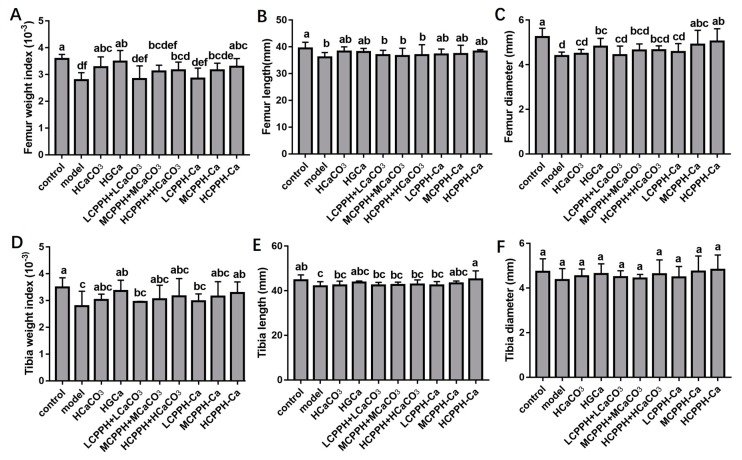
Weight index, length and diameter of femurs and tibias of Ca-deficient rats after treatment with different Ca. Femur weight index (**A**), Femur length (**B**), Femur diameter (**C**), Tibia weight index (**D**), Tibia length (**E**), Tibia diameter (**F**). Note: control, normal group; model, low calcium group; HCaCO_3_, high dosage of CaCO_3_ group; HGCa, high dosage of calcium gluconate group; LCPPH + LCaCO_3_, low dosage of CPPH supplemented with low dosage of CaCO_3_ group; MCPPH + MCaCO_3_, model dosage of CPPH supplemented with model dosage of CaCO_3_ group; HCPPH + HCaCO_3_, high dosage of CPPH supplemented with high dosage of CaCO_3_ group; LCPPH-Ca, low dosage of CPPH-Ca group; MCPPH-Ca, model dosage of CPPH-Ca group; HCPPH-Ca, high dosage of CPPH-Ca group. Data are expressed as the mean ± SD (*n* = 10). One-way ANOVA with Tukey’s test. Different letters indicate significant effect with *p* < 0.05.

**Figure 3:**
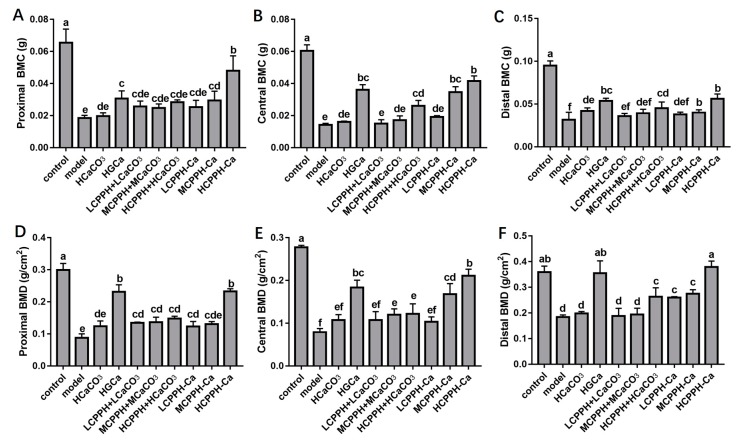
Femurs bone mineral content (BMC) and bone mineral density (BMD) of Ca-deficient rats after oral gavage with different Ca in the experimental period. Proximal BMC (**A**), Central BMC (**B**), Distal BMC (**C**), Proximal BMD (**D**), Centrality BMD (**E**), Distal BMD (**F**). Note: control, normal group; model, low calcium group; HCaCO_3_, high dosage of CaCO_3_ group; HGCa, high dosage of calcium gluconate group; LCPPH + LCaCO_3_, low dosage of CPPH supplemented with low dosage of CaCO_3_ group; MCPPH + MCaCO_3_, model dosage of CPPH supplemented with model dosage of CaCO_3_ group; HCPPH + HCaCO_3_, high dosage of CPPH supplemented with high dosage of CaCO_3_ group; LCPPH-Ca, low dosage of CPPH-Ca group; MCPPH-Ca, model dosage of CPPH-Ca group; HCPPH-Ca, high dosage of CPPH-Ca group. Data are expressed as the mean ± SD (*n* = 10). One-way ANOVA with Tukey’s test. Different letters indicate significant effect with *p* < 0.05.

**Figure 4:**
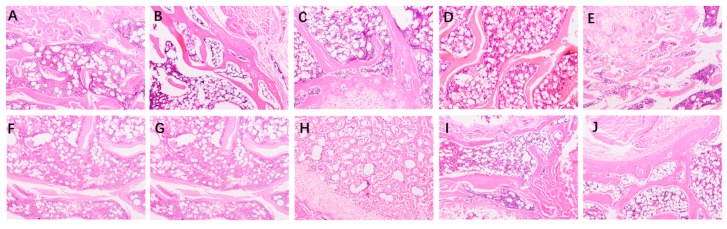
Histopathological analysis of rat kidney tissues in different groups at 100× magnification. control (**A**), model (**B**), HCaCO_3_ (**C**), HGCa (**D**), LCPPH + LCaCO_3_ (**E**), MCPPH + MCaCO_3_ (**F**), HCPPH + HCaCO_3_ (**G**), LCPPH-Ca (**H**), MCPPH-Ca (**I**), HCPPH-Ca (**J**).

To measure the assimilation and metabolism of calcium, femur properties were monitored in the experiment. Figure 2 presents the dry weight (DW) index, length and diameter of femurs and tibias of all rats. At the end of 8 weeks, no significant differences in tibial diameter were observed in any group (Figure 2F) (*p* < 0.05). The femur weight index, femur length, femur diameter, tibial weight index and tibial length of the high dose of CPPH-Ca and HGCa groups were significantly increased compared to the model group (Figure 2A–E) (*p* < 0.05). Meanwhile, there was no statistically significant difference between the HCPPH-Ca group or HGCa group and control group in the femur weight index, femur length, femur diameter, tibial weight index and tibial length (*p* < 0.05). The femur weight and the length of high dose of CaCO_3_, HGCa and the medium and high dose of CPPH-Ca groups were significantly higher compared with the model group (*p* < 0.05), while no significant differences among HCaCO_3_, HGCa and HCPPH-Ca groups were observed (Figure 2A,B,D,E). Particularly, the bone biomechanical parameters of rats in the low, medium and high dose CPPH-Ca groups were higher than those in the CPPH + CaCO_3_ group. Figure 3 indicates that the levels of BMD and BMC at the proximal, central, and distal ends of the femur at the end of 8 weeks were significantly increased in the high dose of CPPH-Ca, HGCa and control groups compared with the model and HCaCO_3_ groups (*p* < 0.05). However, the femur BMD and BMC were significantly different between the high dose of CPPH-Ca and HGCa groups, and the high dose of CPPH-Ca group exhibited a greater effect compared with the HGCa group. The administration of high doses of CPPH-Ca significantly increased the levels of BMD and BMC compared with the high dose of CPPH + CaCO_3_ group at the end of the experiment (8 weeks) (*p* < 0.05) (Figure 3A–F). There was no significant difference in the levels of BMD and BMC of low and medium doses of CPPH + CaCO_3_, while the level of distial BMD reached that of the control group when fed with high doses of CPPH-Ca. H&E staining exhibited the effect of CPPH-Ca on pathological profiles in the femur (Figure 4). According to microarchitectural of femoral necks analysis, the sections of femur tissues in the control group showed normal level (Figure 4A). However, the model group showed abnormal (Figure 4B) bone volume per tissue volume (BV/TV), trabecular thickness (Tb.Th), trabecular number (Tb.No). In the rats fed with CPPH-Ca, we found that the bone volume compared to the tissue volume (BV/TV), trabecular thickness (Tb.Th), trabecular number (Tb.No) was significantly increased, whereas trabecular separation (Tb.Sp) was dramatically decreased. The aforementioned results indicate the beneficial effects of HCPPH-Ca and HCPPH + HCaCO_3_ treatments on the femoral pathology in calcium-deficient rats (Figure 4G,J).

### 2.4. Calcium Balance

**Figure 5:**
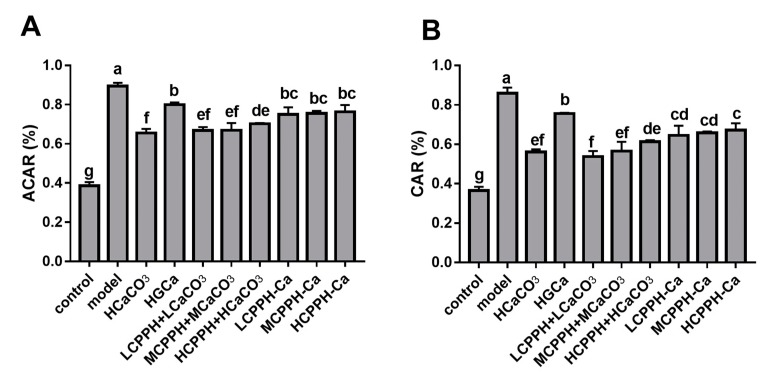
Apparent calcium absorption rate (ACAR) and calcium accumulation rate (CAR) of Ca-deficient rats after oral gavage with different Ca. ACAR (**A**), CAR (**B**). Note: control, normal group; model, low calcium group; HCaCO_3_, high dosage of CaCO_3_ group; HGCa, high dosage of calcium gluconate group; LCPPH + LCaCO_3_, low dosage of CPPH supplemented with low dosage of CaCO_3_ group; MCPPH + MCaCO_3_, model dosage of CPPH supplemented with model dosage of CaCO_3_ group; HCPPH + HCaCO_3_, high dosage of CPPH supplemented with high dosage of CaCO_3_ group; LCPPH-Ca, low dosage of CPPH-Ca group; MCPPH-Ca, model dosage of CPPH-Ca group; HCPPH-Ca, high dosage of CPPH-Ca group. Data are expressed as the mean ± SD (*n* = 10). One-way ANOVA with Tukey’s test. Different letters indicate significant effect with *p* < 0.05.

Figure 5 shows that the oral administration of low, medium and high doses of CPPH-Ca remarkably improved the apparent calcium absorption (ACAR) of rats compared with CaCO_3_ (*p* < 0.05) (Figure 5A), and that these groups had no significant difference in ACAR between the control and HGCa group. Meanwhile, the administration of the low, middle and high doses of CPPH-Ca significantly increased the calcium accumulation rate (CAR) (*p* < 0.05), indicating the beneficial effects of CPPH-Ca treatment on calcium absorption in calcium-deficient rats. Moreover, the CAR in the low, medium and high doses CPPH-Ca groups were significantly higher compared with the HCaCO_3_ group (*p* < 0.05). Rats in the HCPPH-Ca group showed significantly higher ACAR and CAR compared with the HCPPH + HCaCO_3_ group (*p* < 0.05). The ACAR and CAR of the CPPH + CaCO_3_ group at different doses were not significantly different from those of the corresponding CPPH-Ca groups (*p* < 0.05).

### 2.5. Gene Expression of Corresponding Receptors in the Kidney of the Rats 

**Figure 6:**
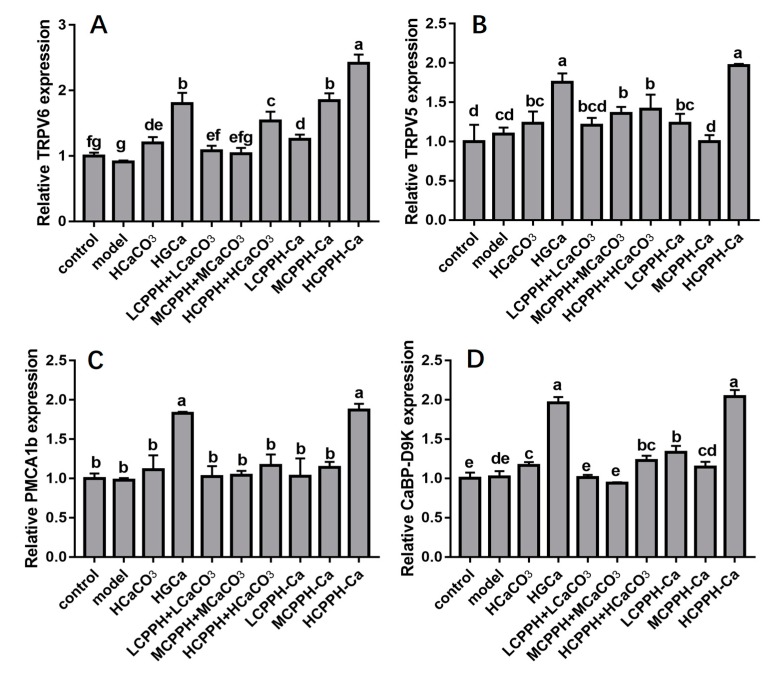
mRNA expression levels of genes involved in calcium-promoting mechanism as determined using real-time PCR. Transient receptor potential cation V6 (TRPV6) (**A**), Transient receptor potential cation V5 (TRPV5) (**B**), calcium-binding protein-D9k (CaBP-D9K) (**C**), plasma membrane Ca-ATPase (PMCA1b) (**D**). Note: control, normal group; model, low calcium group; HCaCO_3_, high dosage of CaCO_3_ group; HGCa, high dosage of calcium gluconate group; LCPPH + LCaCO_3_, low dosage of CPPH supplemented with low dosage of CaCO_3_ group; MCPPH + MCaCO_3_, model dosage of CPPH supplemented with model dosage of CaCO_3_ group; HCPPH + HCaCO3, high dosage of CPPH supplemented with high dosage of CaCO_3_ group; LCPPH-Ca, low dosage of CPPH-Ca group; MCPPH-Ca, model dosage of CPPH-Ca group; HCPPH-Ca, high dosage of CPPH-Ca group. Data are expressed as the mean ± SD (*n* = 10). One-way ANOVA with Tukey’s test. Different letters indicate significant effect with *p* < 0.05.

We examined the expressions of genes involved in calcium absorption in the kidney to assess the molecular mechanisms of CPPH-Ca in regulating the calcium absorption mechanism (Figure 6). Our data revealed that there was a significant increase in most genes at the mRNA level responsible for calcium absorption in kidney of CaBP-D9k, TRPV6 and TRPV5 by the HGCa and the high dose of CPPH-Ca treatment when compared with the model group (*p* < 0.05) (Figure 6A,B,D). PMCA1b is located on the basolateral membrane and plays a role in the extrusion of calcium. We found that the PMCA1b expression was significantly elevated in the HCPPH-Ca group compared with the control group (*p* < 0.05) (Figure 6C). Collectively, these results indicated that HCPPH-Ca promoted calcium absorption in the kidney of calcium-deficient rats, suggesting that calcium supplementation of HCPPH-Ca enhanced calcium absorption, activated calcium transport channels and up-regulated intracellular calcium buffering genes.

### 2.6. CPPH-Ca Modulates Caecal Microbiota Composition of Calcium-Deficient Rats

**Figure 7:**
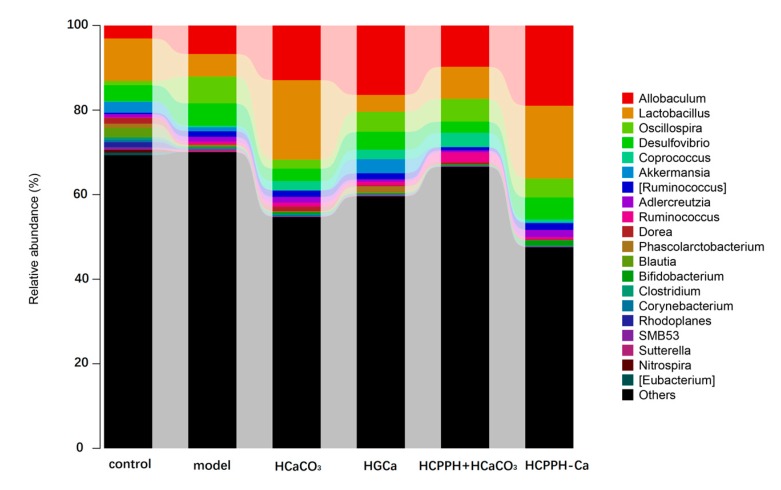
Changes in the bacterial composition of rat intestinal contents according to different genera. Composition of gut microbiota at the genus level. Note: control, normal group; model, low calcium group; HCaCO_3_, high dosage of CaCO_3_ group; HGCa, high dosage of calcium gluconate group; LCPPH + LCaCO_3_, low dosage of CPPH supplemented with low dosage of CaCO_3_ group; MCPPH + MCaCO_3_, model dosage of CPPH supplemented with model dosage of CaCO_3_ group; HCPPH + HCaCO_3_, high dosage of CPPH supplemented with high dosage of CaCO_3_ group; LCPPH-Ca, low dosage of CPPH-Ca group; MCPPH-Ca, model dosage of CPPH-Ca group; HCPPH-Ca, high dosage of CPPH-Ca group. T-test was used to calculate significant differences between group.

To identify the effects of HCPPH-Ca on the compositional distribution of caecal microbiota, we investigated the dominant microbial populations in the other groups (Figure 7) using high-throughput sequencing (HTS) technology. Additionally, the V3–V4 regions of the 16S rRNA gene from fecal samples were sequenced using the Illumina MiSeq platform. At the genus level of metagenomic analysis, the calcium deficiency induced by low calcium diet changed the composition of the intestinal microbiota when compared with the control group. However, the gut microbiota populations of the HCPPH-Ca group recovered. In this study, *Allobaculum, Lactobacillus, Oscillospira, Desulfovibrio, Coprococcus, Oscillospira, Akkermansia, Adlercreutzia, Dorea, Blautia, Ruminococcus, Bifidobacterrium* and *Clostridium* were the prevailing genera in different groups. Through 8 weeks of HCPPH-Ca treatment, the relative abundances of these bacteria were significantly altered, and *Allobaculum, Lactobacillus* and *Ruminococcus* were the most prominently enriched ones after HCPPH-Ca treatment at the genus level. In addition, the abundance of *Coprococcus* was reduced in the HCPPH-Ca group compared with the model group. These results suggested that low-calcium diets could dysregulate the distribution of gut microbiota, while HGCa treatment might have the ability to restore the ecological imbalance of the intestinal flora, maintaining its healthy composition.

### 2.7. Correlations of Biochemical Data and Key Phylotypes of Caecal Microbiota

**Figure 8:**
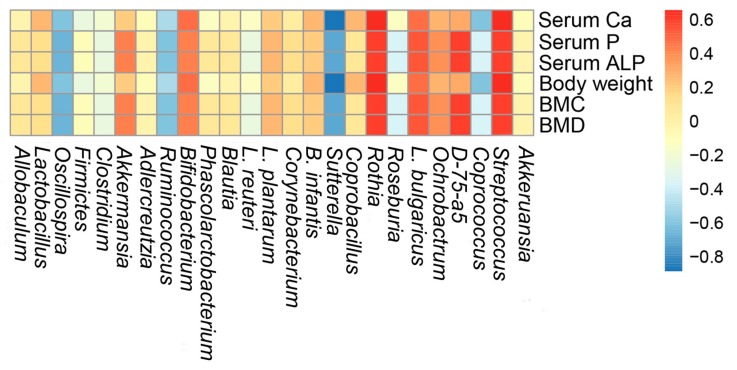
Heatmap of Spearman’s correlation between caecal microbiota of significant differences and biochemical indexes. The depth of the color corresponds the extent of relevance between caecal microbiota and biochemical indexes. (FDR adjusted *p* < 0.05).

In the present study, we explored the interactive features between the calcium absorption and gut microbiota during the calcium deficiency-induced development. The correlation between the composition of gut microbiota and biochemical indicators induced by HCPPH-Ca was also assessed by Spearman’s algorithm (Figure 8). The microbes, including *Lactobacillus*, *Rothia*, *Streptococcus* and *Turicibacter*, showed a positive correlation with abnormal parameters, such as serum Ca and body weight, while *Sutterella* was negatively correlated with the serum Ca and body weight. Interestingly, *D-75-a5*, *Akkeruansia*, *Rothia* and Streptococcus were positively correlated with serum P, serum Ca levels and body weight. These results indicated that these bacteria played an important role in the beneficial effects of HCPPH-Ca.

## 3. Discussion

To provide more information on the binding of metal ions with organic ligand groups of peptide, the FTIR spectra are shown (Figure S2).The specific FTIR absorption peak of amide-A stretching vibration had significant fluctuations at 3410 cm^−1^, which might be attributed to the substitution of N-OH bonds (hydrogen bonds) with Ca-N bonds after calcium chelation [51,52]. The amide-I vibration and amide-II vibration were important vibrational modes of amides. The amide-I vibration is primarily caused by the stretching of C=O bonds and amide-II vibration is assigned to deformation of N-H bonds and stretching of C–N bonds [13,14]. The absorption band of FY at 1642 cm^−1^ for the amide I band shifted to a higher frequency (1650 cm^−1^) when chelating with calcium, showing that the -COO- group participated in the covalent combining reaction with the metal cations [13]. After chelation, the spectrum shifted towards high-frequency wavenumbers (3500–2800 cm^−1^), indicating that the dipole field effect or induced effect led to the electron cloud density and frequency increase. Strong absorption peaks at 1600 cm^−1^ and 1300 cm^−1^ for the amide I band showed that the -COO- group participated in the covalent combination reaction with the metal cations [53]. The maximum absorption peak of the CPPH was at around 230 nm, and the maximum absorption peak of the CPPH-Ca was transferred at around 223 nm. This showed that the chromophore groups (C=O, -COOH) and auxochrome groups (-OH, -NH_2_) induced intensity changes and red shift in the ultraviolet spectrum when the CPPH was combined with calcium ions to form a chiral spatial structure [54]. Therefore, structural characterization of CPPH-Ca showed that CPPH and Ca^2+^ were combined to form a new substance.

Low-calcium diets may cause a significant reduction in body weight and changes in the serum parameters, gut microbiota disturbance and intestinal barrier dysfunction as well as osteoporosis, hypertension and rickets [55,56]. Therefore, various types of calcium-fortified medicines and foods have come onto the market. However, calcium deficiency is still widespread due to insufficient absorption of the intake calcium and low bioavailability [57,58,59,60]. Currently, organic calcium (especially the peptide-calcium complex) as a new type of calcium supplement has become a hot research topic, due to its good therapeutic effects in clinical practice [61,62]. In the present study, we investigated whether CPPH + CaCO_3_ and CPPH-Ca promoted calcium absorption and how such an effect might impair kidney gene expression and gut microbiota. Rats fed with high doses of CPPH-Ca increased serum Ca and P concentration, and decreased serum ALP level. The results indicated that high doses of CPPH-Ca increased serum Ca and P concentrations, which rose to values comparable to those of the control group [4]; this was consistent with previously reported studies [63,64]. Meanwhile, CPPH-Ca may influence organism growth. These results of this observation could be the ability of a high dose of CPPH-Ca to increase the calcium retention and prevent mineral loss [4]. ALP play an important role in the process of bone calcification; it will increase when calcification is abnormal [64]. High serum ALP level may interfere with calcium absorption [65,66,67,68]. The results illustrated that the high dose of CPPH-Ca treatment could restore calcification to a normal level, and that calcium in HCPPH-Ca was more easily absorbed, calcified and its effect was better compared with inorganic calcium, even in the HGCa.

Femoral properties are the ideal monitoring indicators in the calcium supplementation experiment because they are sensitive to Ca assimilation and metabolism [63]. The consumption of low calcium (model) obviously decreased the dry weight (DW) index, length and diameter of femurs and tibias, indicating that calcium deficiency resulted in calcification of rat bone, which were consistent with a previous study [63]. In our study, the high dose of CPPH-Ca supplementation significantly alleviated the levels of femoral and tibial weight index, length and diameter in low-calcium diet-fed rats during eight-weeks, suggesting that HCPPH-Ca had positive effects on calcium absorption and bone calcification. Bone content (BMC) and bone mineral density (BMD) are used to assess the bone strength and quality of bone [62]. The loss of bone mass is accompanied by increased bone remodeling, evidenced by elevated serum ALP activity. Interestingly, our results illustrated that HCPPH-Ca treatment could effectively improve bone index parameters, and more effective absorption and utilization of calcium were found in rats fed HCPPH-Ca compared with inorganic calcium and even HGCa [67]. BMD and BMC are the gold standards for the evaluation of low-calcium deficiency risk [66]. In addition, the BMD of femur and tibia were markedly reduced in the model and HCaCO_3_ groups compared with the HCPs-Ca, HGCa and control groups. The 8-week treatment of HCPPH-Ca improved BMD and BMC and prevented the bone loss induced by calcium deficiency. As previously described, calcium deficiency is a metabolic bone disease characterized by reduced BMD and BMC. BMC and BMD in rats can be improved by supplementation of bovine and caprine cheese [68]. In addition, these changes of calcium deficiency phenotypes might be associated with femoral mineralization. In this study, low-calcium diets could cause morphological changes of trabecular bone and femoral mineralization of rats [68]. Histopathological analyses revealed visible differences of femur tissue structure in these ten groups. The histopathological femur was improved by HCPPH-Ca supplementation, for which the result was similar to previous research [66]. Meanwhile, the calcium bioavailability of a body is usually increased in cases of severe calcium deficiency. A previous study showed that effective calcium supplementation can improve the ACAR and CAR [57]. In our experiment, after 8 weeks, HCPPH-Ca treatment significantly elevated ACAR and CAR compared with the HCPPH + HCaCO_3_ and HCaCO_3_ groups (*p* < 0.05). With long-term low-calcium intake or bioavailability, the calcium level in the circulation decreases to below normal levels. Therefore, in order to maintain the blood calcium level, calcium is taken from the bone into the blood. These findings confirmed that HCPPH-Ca could be used as an effective calcium supplement in ameliorating the low-calcium diet-induced effects.

To further understand the molecular mechanism of HCPPH-Ca underlying the calcium re-absorption, we examined the expressions of the genes involved in kidney at the mRNA level, including CaBP-D9k [37], TRPV6 [36] and TRPV5 [35], as well as the expression of PMCA1b [69]. In the kidney, TRPV5, TRPV6, PMCA1 and CaBP-9k were principally expressed in the basolateral layer of the distal and proximal convoluted tubules. Several calcium transporters (CaBP-D9k, and PMCA1) have already been shown to express in the convoluted tubules [64], as has the function of calcium transporters transport active calcium in the kidney. When the body is deficient in calcium, calcium ions are removed from kidneys through calcium transport genes such as TRPV5, TRPV6, CaBP-D9k, and PMCA1 [70]. Calcium supplementation can help to prevent serious disorders such as hypercalcemia and hypocalcemia [64]. TRPV6 is an apical calcium entry channel in the kidney in general and particularly is to renal transcellular Ca^2+^ re-absorption, which regulates two separate active transcellular pathways for Ca^2+^ absorption [70] Importantly, co-localization of TRPV5 and TRPV6 in the kidney may have significant functional relevance, because it was recently shown that TRPV5 and TRPV6 can form heterotetrameric Ca^2+^ channels with distinct functionality [70]. The pathway regulated by TRPV6 is dependent on CaBP-D9k and PMCA1 for Ca^2+^ absorption [36]. In addition, TPPV6 is activated when the hyperpolarization of apical membrane occurs. Moreover, TPPV6 can be modulated by vitamin D3 along with CaBP-D9k and PMCA1b [47,48], indirectly indicating that HCPPH-Ca is the active ingredient responsible for calcium absorption. This process is possibly regulated by interacting with these calcium transporters and enzymes involved in intestinal calcium absorption and increasing parathyroid hormone [35,36,37,38].

The gut microbial community plays an important role in maintaining the normal physiological functions of the human body [71]. Therefore, we compared the caecal microbiota of rats in different groups to elucidate the precise underlying mechanism of improved calcium absorption by HCPPH-Ca. Our results showed that HCPPH-Ca treatment significantly changed the relative abundances of gut microbiota induced by low-calcium diets, including *L. reuteri*, *L. plantarum*, *Firmicutes*, *L. bulgaricus*, *Streptococcus thermophilus* and *Lactobacillus* (*p* < 0.05). *Bifidobacterium* and *L. reuteri* showed a positive correlation with the levels of serum Ca, ALP, BMC and BMD. Previous work has demonstrated that the beneficial effect of *L. reuteri* on bone health is dependent on immunomodulation of key pathways involved in osteoclastogenesis, estrogen signaling [71,72,73] and BMD [74,75,76,77,78,79]. Different strains of *Lactobacillus* and *Bifidobacterium* possess anti-inflammatory effects, which can improve vitamin D absorption and diminish osteoclast differentiation, thereby preventing the ovariectomy-induced bone loss in mice [80,81,82]. Treatment of either *Lactobacillus rhamnosus GG* (LGG) or the commercially available probiotic supplement reduces gut permeability in mice, inhibits the intestinal and bone marrow inflammation, and completely prevents bone loss after sex steroid deprivation, leading to both down-regulation of bone resorption markers and up-regulation of bone formation markers [83,84,85]. *Lactobacillus* salivarius significantly increases cellular calcium uptake [86,87]. Several studies have shown that administration of probiotics, such as *Lactobacillus (L) reuteri, L. plantarum, Bifidobacterium (B) longum*, or mixtures of several species, can exert a protective effect against bone loss in ovariectomized mice. Moreover, HCPPH-Ca reduced the abundance of *Firmicutes* and enhanced the abundance of Bacteroidetes in caecal contents. UTOHERE It is necessary to determine whether other gut microbiome play a causal role in bone calcium absorption in CPPH-Ca [88]. Taken together, we offered convincing evidence for the potential use of CPPH-Ca in calcium deficiency and showed that the gut microbiota played a potent regulatory role in attenuating metabolic abnormalities. Figure S3 illustrates the mechanism by which HCPPH-Ca promoted calcium absorption. HCPPH-Ca could promote calcium absorption partially through regulating specific gut microbiota and modulating the expressions of the calcium absorption-related genes in the kidney. Therefore, HCPPH-Ca could be beneficially used to promote the calcium absorption and reduce the risk of calcium deficiency.

## 4. Materials and Methods

### 4.1. Preparation of CPPH, CPPH-Ca and HPLC-MS/MS Analysis

*Chlorella pyrenoidosa* powder was purchased from King Dnarmsa *Spirulina* Co., Ltd. (Fuqing, China). In order to get the CPPH and CPPH-Ca, CPP was first prepared. Briefly, *Chlorella pyrenoidosa* powder was dissolved in 0.2 M NaOH solution at a concentration of 3.3%. CPP was extracted at 60 °C for 60 min. The extract was centrifuged at 4500 rpm for 10 min, and then the pH of supernatant was adjusted to 3.0 by 4 M HCl solution and allowed to stand for 30 min. The precipitate was collected by centrifugation at 5000 rpm for 10 min and freeze-dried for further enzymatic hydrolysis. The mixture of CPP powder and ultra-pure water (powder: water, 1:30; W/W) was hydrolyzed by neutral protease (3%, neutral protease: substrate, protein basis) at pH 7.5 for 6 h at 40 °C. and the hydrolyzed solution was incubated in boiling water for 10 min to inactivate the enzyme. The pH of the mixture was adjusted to neutral, followed by centrifugation at 5000 rpm for 10 min. The supernatant was freeze-dried, and CPPH was yielded. CPPH-Ca was obtained by mixing 4% CPP solution with CaCl_2_ (4.5 g/100 g peptide) at pH 9, followed by incubation at 50 °C for 30 min. Thereafter, the free calcium was removed with a semi-permeable membrane with a cut-off of 100 Da (Thermo Fisher Scientific Inc., Waltham, UK). Subsequently, 95% ethanol was added, and the mixture was allowed to stand at 25 °C for 24 h, followed by centrifugation at 4500 rpm for 10 min. The supernatant was discarded, and the precipitate was collected and washed with 70% ethanol for three times to remove the superfluous Ca^2+^. Finally, the precipitate was freeze-dried and labeled as CPPH-Ca. The obtained CPPH-Ca was stored in a desiccator for further analysis. Peptide contents of CPPH and CPPH-Ca were determined by Testing Center of Fuzhou University. The calcium concentration of CPPH-Ca was determined with an atomic absorption spectrophotometer (AA-6300C, Shimadzu, Kyoto, Japan). The components of CPPH were determined on an HPLC-MS/MS, and the analytical column was a Waters BEH C18 column (1.7 µm, 2.1 × 50 mm) (Macherey-Nagel, Düren, Germany) as previously described [89].

### 4.2. Structural Characterization of Peptide-Calcium Chelate by Ultraviolet Spectroscopy and FTIR Analysis

The ultraviolet spectra of CPPH and CPPH-Ca were monitored by an ultraviolet spectrophotometer (UV-2600, UNICO Instrument Co. Ltd., Shanghai, China) in the wavelength range of 190–400 nm. The mixture of lyophilized sample (1 mg) and dried KBr (100 mg) were loaded onto a Fourier transform infrared spectrometer. Infrared spectral scans of wavelengths from 4000 cm^−1^ to 400 cm^−1^ were recorded by FTIR spectroscopy infrared spectrophotometer (360 Intelligent, Thermo Nicolet Co., Madison, WI, USA).

### 4.3. Animals 

Male SD rats (3 weeks old) were purchased from Wu’s Experimental Animal Company (Fuzhou, China). The animals were bred in stainless steel wire-bottomed cages under hygienic standard environmental conditions (23 ± 1 °C; humidity of 60 ± 5%; 12 h dark/light cycle). The rats were given free access to commercial food, which was prepared according to the AIN-93 (normal diet: 5000 mg Ca/kg; low-calcium diet: 1000 mg Ca/kg), and tap water. The AIN-93 diet was provided by Trophic Animal Feed High-Tech Co., Ltd. (Nantong, China). All animal-related protocols were in accordance with laboratory animal welfare ethics and daily animal care guidelines, and approved by the Ethics Review Committee of the College of Food Science, Fujian Agriculture and Forestry University (No. FS-2018-008). After animals were acclimatized for 1 week, 100 rats were randomly and evenly divided into 10 groups (Table 2). The rats in the control group were fed with the normal diet. Remaining rats were fed with a low-Ca diet, which were randomly assigned into 9 groups of 10 rats each, 9 groups as follows: model group, HCaCO_3_ group, LCPPH + LCaCO_3_ group, MCPPH + MCaCO_3_ group, HCPPH + HCaCO_3_ group, or LCPPH-Ca group, MCPPH-Ca group and HCPPH-Ca group.

### 4.4. Sample Collection 

During a 4-week experimental period, body weight, food intake, the amounts of feces and urine were recorded daily. After the 8-week experiment, all rats were starved for 12 h, anesthetized (1 mg kg^−1^ pentobarbital sodium) and dissected to obtain blood specimens. Blood was collected from the heart, centrifuged at 3000 rpm for 10 min at 4 °C and then stored at −80 °C until further analysis. Kidneys were weighed and cut into several sections. One part of kidney was stained by haematoxylin and eosin (H&E) for histopathological analysis. Another part of kidney was stored at −80 °C until further analysis of the mRNA expressions involved in calcium absorption metabolism. The samples of fresh caecal contents were also immediately collected before snap-freezing in liquid nitrogen and stored at −80 °C. Finally, the left and right femurs and tibias of each rat were dissected and cleaned of soft tissues. The left femur was stored in 4% paraformaldehyde for histopathological analysis.

### 4.5. Serum Calcium, Phosphorus Content and ALP Activity

Levels of serum calcium, phosphorus and ALP were determined using the corresponding assay kits (Nanjing Jiancheng Bioengineering Institute, Nanjing, China).

### 4.6. Bone Index Parameter and Histomorphometry Analysis

The left femurs of all rats were measured with a digital caliper (Exploit, Beijing, China). Then, femurs were dried in an oven at 105 °C overnight and weighed on an analytical balance (ML204, METTLER TOLEDO, Zurich, Switzerland). Dry weight index (DW index) was calculated according to the equation: DW index (10^−3^) = Dry weight × 1000/Body weight. Bone mineral content (BMC) and bone mineral density (BMD) were assayed by the PIXImus dual energy X-ray absorptiometry method (LUNAR Corporation, Mississauga, ON, Canada) as described by Fonseca and Ward [90]. The femurs tissues were removed from each mice and samples were subsequently fixed in 4% 128 (v/v) paraformaldehyde/PBS then treated with ethanol solution. After that, all femur samples were decalcified in 10% EDTA (pH 7.4) for 3 weeks and were embedded in paraffin, the slices of distal femur were sectioned at 5 mm then were stained with H&E to observe the morphological changes under the high magnification of an optical microscope with high magnification (Nikon Eclipse TE2000-U, Nikon, Japan) [91].

### 4.7. Calcium Balance Study

Food consumption was recorded, and the urine and feces were measured and collected daily. The calcium balance experiment was conducted. After sample collection, the urine was immediately centrifuged at 3000× *g* for 10 min, and the supernatant was obtained. Fresh fecal samples were collected immediately after defecation and freeze-dried. The calcium contents of urine and feces were determined by a flame atomic absorption spectrometer (AA-6300C, Shimadzu). The ACAR and CAR were calculated as follows:

ACAR (%) = (Ca intake − feces Ca) × 100/Ca intake(1)

CAR (%) = (Ca intake − feces Ca − urine Ca) × 100/Ca intake(2)

### 4.8. RT-qPCR Analysis

Total RNA was extracted from the frozen kidney tissues by using TRIzol reagent (Invitrogen, Carlsbad, CA, USA). Purified RNA was reversely transcribed into cDNA using PrimeScript™ RT reagent Kit with gDNA Eraser (Takara, Kusatsu, Japan). The expressions of TRPV6, TRPV5 and CaBP-D9K were assessed by RT-qPCR using the SYBR^®^ Premix Ex Taq™ II (Takara, Kusatsu, Japan) on an AB7300 Real-Time PCR system (Applied Biosystems, Foster City, CA, USA). GAPDH was selected as the housekeeping gene. The primer sequences were as follows: TRPV6, F: 5′-GCACCTTCGAGCTGTTCC-3′, R: 5′-CAGTGAGTGTCGCCCATC-3′; TRPV5, F: 5′-CGAGGATTCCAGATGC-3′, R: 5′-GACCATAGCCATTAGCC-3′; PMCA1b, F: 5′-TTCAGGTACTCATGTGATGGAAGG-3′, R: 5′-CAGCCCCAAGCAAGGTAAA-3′; CaBP-D9K, F: 5′-GACCTCACCTGTTCCTGTCTG-3′, R: 5′- GCTCCTTCTTCTGGCTTCATT-3′; GAPDH, F: 5′-TGACTTCAACAGCGACACCCA -3′, R: 5′-CACCCTGTTGCTGTAGCCAAA-3′. Briefly, after an initial denaturation step at 95 °C for 30 s, the amplifications were carried out with 40 cycles at a melting temperature of 95 °C for 5 s, an annealing temperature of 60 °C for 30 s, and an extension temperature of 72 °C for 30 s. The data were analyzed using Rotor-gene Q software ver. 1.7 (Qiagen). Relative gene expressions were calculated by the comparative 2^−ΔΔCt^ method.

### 4.9. Extraction of Caecal Genomic DNA for High Throughput Sequencing

Gut microbiota analysis was performed on ten samples per group. Metagenomic DNA was extracted from the caecal contents of the rats using a PowerSoil DNA Isolation Kit (MO BIO Laboratory Inc., Carlsbad, CA) according to the manufacturer’s instructions. The 16S rRNA gene (V3-V4 hypervariable regions) from the caecal microbiota was amplified using specific primers (forward primer 5′-CCTACGGRRBGCASCAGKVRVGAAT-3′ and reverse primer 5′-GGACTACNVGGGTWTCTAATCC-3′) [92]. Sequencing was performed using a 2 × 300 paired-end (PE) configuration, and the amplification bias caused by a non-official barcode was avoided. Finally, the image analysis and base calling were conducted on the MiSeq reagent kit v2 (300 cycles) by the MiSeq platform (Illumina, Inc., San Diego, CA, USA). The initial taxonomy analysis was performed on Illumina’s BaseSpace cloud computing platform.

### 4.10. Bioinformatics Analysis

High-quality sequences were assigned to samples according to barcodes. The valid sequences were denoised in order to assess the species diversity. Results were generated using Usearch (Version 7.1, http://drive5.com/uparse/) with a disagreement of 3% [93].

### 4.11. Statistical Analysis

Data for each group were expressed as mean ± SD (*n* = 10). Statistical significance was measured using one-way analysis of variance (ANOVA) with Tukey’s test. Statistical significance was expressed by a p-value of less than 0.05. Spearman’s rank correlation coefficient was used to assess the correlation between gut microbiota and lipid metabolic parameters.

## 5. Conclusions

Collectively, the structural characterization of CPPH-Ca showed that CPPH and Ca^2+^ were combined to form a new substance. This study indicated that the HCPPH-Ca exerted its promotive effects on calcium absorption in the kidney by influencing relational gene expressions, enhancing bone tissues in rapidly growing rats, improving calcium absorption utilization and regulating gut microbiota. Additionally, HCPPH-Ca could improve femoral morphological abnormalities. Moreover, HCPPH-Ca treatment exerted promotive effects on calcium absorption by up-regulating TRPV6, TRPV5, CaBP-D9K and CMBP1b signaling pathways in kidney and affecting the composition of gut microbiome induced by low-calcium diets. Meanwhile, HCPPH-Ca also had beneficial effects on bone tissues in rats compared with the inorganic calcium. Taken together, our study, for the first time, clarified the new potential therapeutic role of HCPPH-Ca. However, in the study, bacterial 16S rRNA gene sequencing could not be fully characterized to assign accurate classification information beyond the genus level due to the limited read length of the Illumina MiSeq platform. Therefore, future studies should further explore changes in gut flora at the species level.

## Figures and Tables

**Table 1:** Changes in the body weight of rats in the different groups during the experimental period.

Groups	Weight (g)			
	0 Weeks	4 Weeks	8 Weeks	Weight Gain
Control	120.04 ± 4.66^a^	303.62 ± 10.43^a^	371.21 ± 14.01^a^	245.13 ± 9.00^a^
Model	125.19 ± 4.79^a^	267.32 ± 5.62^b^	308.56 ± 4.58^e^	189.36 ± 8.33^f^
HCaCO_3_	120.39 ± 9.22^a^	269.63 ± 14.27^b^	326.33 ± 19.47^bcd^	211.42 ± 9.63^bcd^
HGCa	122.70 ± 2.19^a^	271.54 ± 13.35^b^	331.92 ± 11.43^bc^	221.79 ± 10.96^bc^
LCPPH + LCaCO_3_	122.443 ± 4.75^a^	266.95 ± 11.27^b^	320.97 ± 14.36^cde^	196.53 ± 11.53^ef^
MCPPH + MCaCO_3_	119.72 ± 5.43^a^	271.54 ± 17.60^b^	328.01 ± 24.35^bcd^	208.23 ± 21.50^cde^
HCPPH + HCaCO_3_	123.94 ± 4.76^a^	269.85 ± 12.52^b^	333.47 ± 17.22^bc^	214.70 ± 11.34^bcd^
LCPPH-Ca	123.34 ± 4.49^a^	267.35 ± 9.50^b^	325.72 ± 20.89^bcd^	202.38 ± 17.97^def^
MCPPH-Ca	121.65 ± 4.63^a^	269.20 ± 6.67^b^	332.21 ± 11.45^bc^	204.33 ± 15.22^de^
HCPPH-Ca	121.85 ± 7.19^a^	270.62 ± 13.87^b^	340.02 ± 20.01^a^	223.41 ± 13.03^b^

Note: control, normal group; model, low calcium group; HCaCO_3_, high dosage of CaCO_3_ group; HGCa, high dosage of calcium gluconate group; LCPPH + LCaCO_3_, low dosage of CPPH supplemented with low dosage of CaCO_3_ group; MCPPH + MCaCO_3_, model dosage of CPPH supplemented with model dosage of CaCO_3_ group; HCPPH + HCaCO_3_, high dosage of CPPH supplemented with high dosage of CaCO_3_ group; LCPPH-Ca, low dosage of CPPH-Ca group; MCPPH-Ca, model dosage of CPPH-Ca group; HCPPH-Ca, high dosage of CPPH-Ca group. Data are expressed as the mean ± SD (*n* = 10). One-way ANOVA with Tukey’s test. Different letters indicate significant effect with *p* < 0.05.

**Table 2:** Oral administration dosages of peptides and Ca for each group.

No.	Group	n	Given Dosage of N Content and Ca, mg/kg bw
1	Control	10	Deionized water
2	Model	10	Deionized water
3	HCaCO_3_	10	Ca 119.97
4	HGCa	10	Ca 119.97
5	LCPPH + LCaCO_3_	10	CPPH 465 + Ca 39.99
6	MCPPPH + MCaCO_3_	10	CPPH 930 + Ca 79.98
7	HCPPH + HCaCO_3_	10	CPPH1395 + Ca 119.97
8	LCPPH-Ca	10	Ca 39.99
9	MCPPH-Ca	10	Ca 79.98
10	HCPPH-Ca	10	Ca 119.97

Note: Control, normal group; model, low-calcium group; HCaCO_3_, high dosage of CaCO_3_ group; HGCa, high dosage of calcium gluconate group; LCPPH + LCaCO_3_, low dosage of CPPH and low dosage of CaCO_3_ group; MCPPH + MCaCO_3_, middle dosage of CPPH and middle dosage of CaCO_3_ group; HCPPH + HCaCO_3_, high dosage of CPPH and high dosage of CaCO_3_ group; LCPPH-Ca, low dosage of CPPH-Ca group; MCPPH-Ca, middle dosage of CPPH-Ca group; HCPPH-Ca, high dosage of CPPH-Ca group.

## Supplementary Materials

The following are available online at https://www.mdpi.com/1660-3397/17/6/348/s1, Figure S1: UV spectra of CPPH and CPPH-Ca over the wavelength range from 190 to 400 nm, Figure S2: Fourier transform infrared (FTIR) spectra of CPPH and CPPH-Ca chelate in the regions from 4000 to 400 cm^−1^, Figure S3: summary of the mechanism underlying the preventive effects of CPPH-Ca on calcium absorption, Table S1: peptide profile of CPPH identified by HPLC-MS/MS.
