# Supplementary figures and images for: Effect of Chlorella Pyrenoidosa Protein Hydrolysate-Calcium Chelate on Calcium Absorption Metabolism and Gut Microbiota Composition in Low-Calcium Diet-Fed Rats
Source: Mar Drugs. 2019 Jun 11;17(6):348. doi: 10.3390/md17060348 (PMC6628084; doi:10.3390/md17060348)

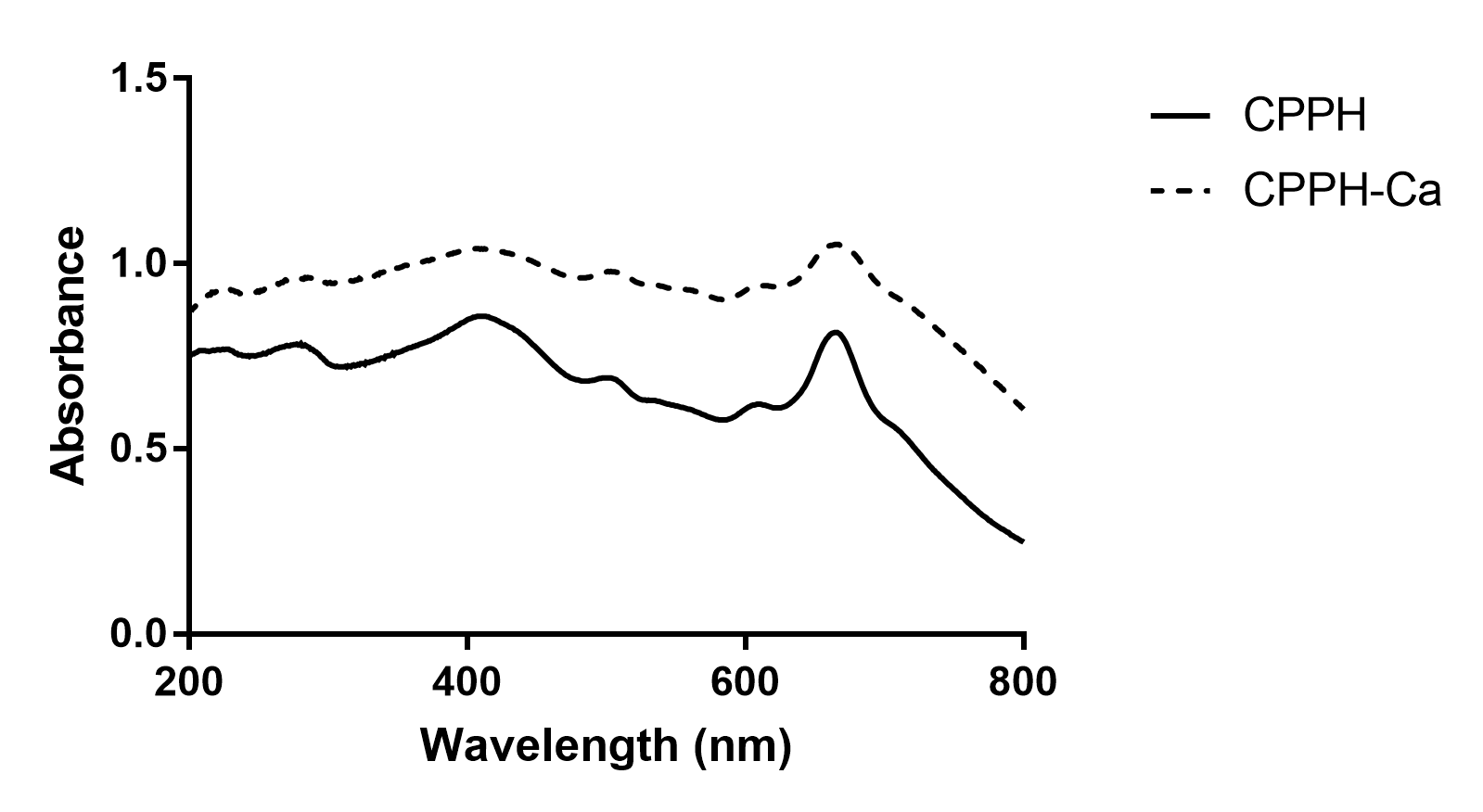

Supplement: Supplementary file 1 [file marinedrugs-17-00348-s001.zip › Supplemental files/Figure S1.tif]

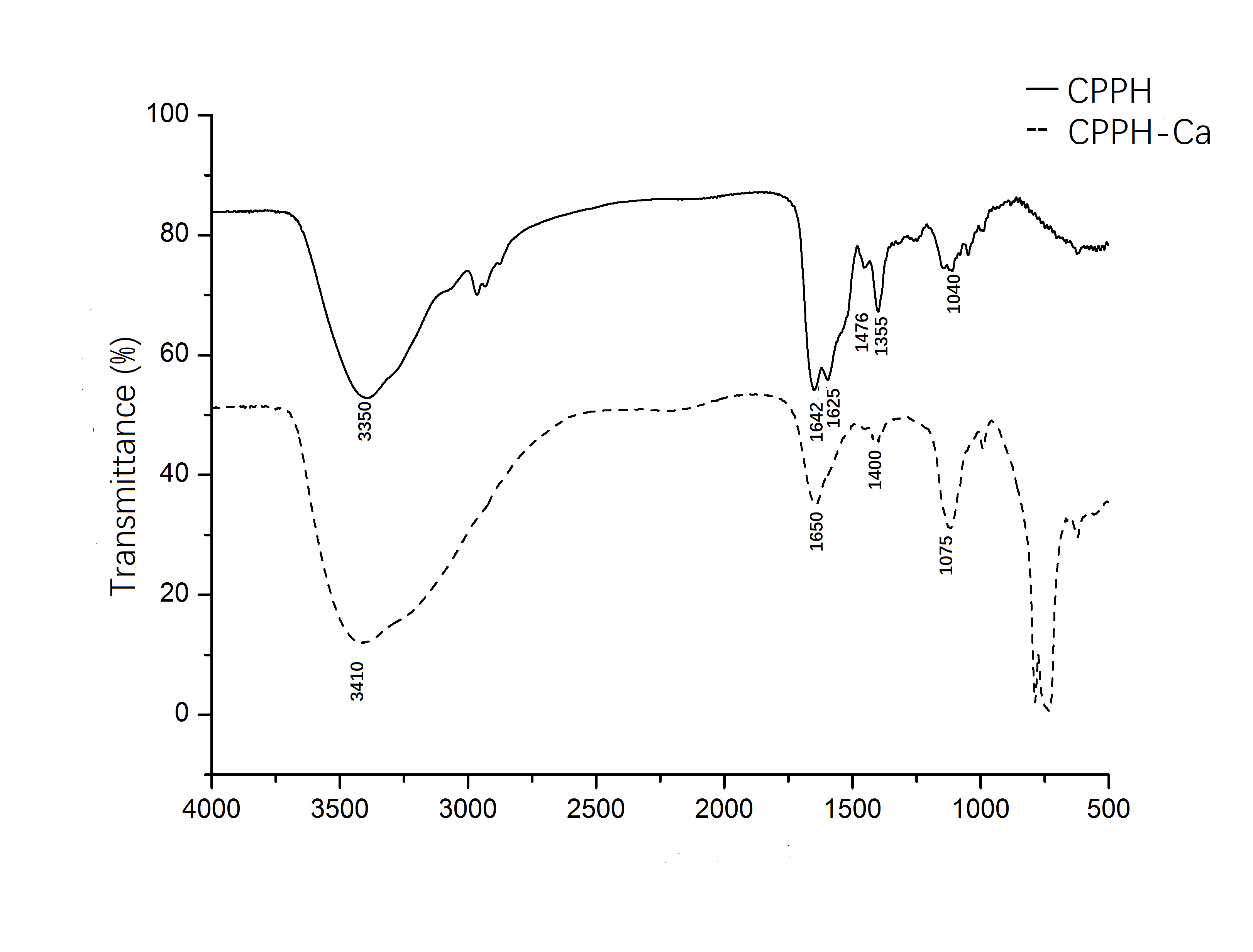

Supplement: Supplementary file 1 [file marinedrugs-17-00348-s001.zip › Supplemental files/Figure S2.tif]

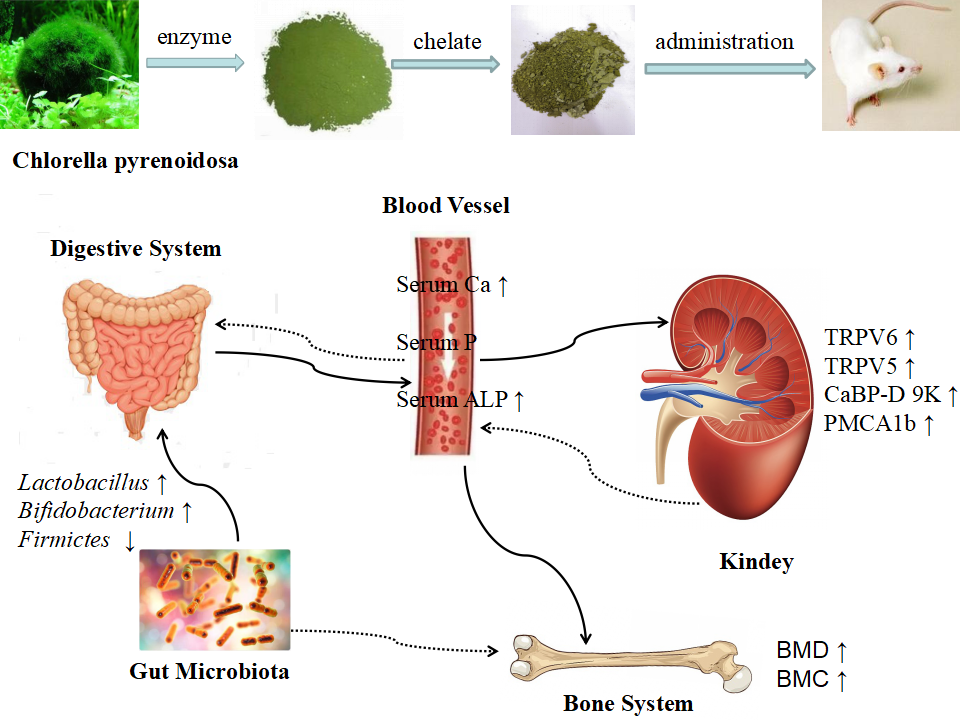

Supplement: Supplementary file 1 [file marinedrugs-17-00348-s001.zip › Supplemental files/Figure S3.tif]
